# Microglia in the neurogenic niche: emerging roles of immunometabolism

**DOI:** 10.3389/fnins.2026.2007175

**Published:** 2026-10-22

**Authors:** Danping Liu, Yunjun Li, Yan Liu

**Affiliations:** 1 Hubei Provincial Hospital of Traditional Chinese Medicine, Affiliated Hospital of Hubei University of Chinese Medicine, Wuhan, China; 2 Wuhan Hospital of Traditional Chinese Medicine, Wuhan,China; 3 Hubei University of Chinese Medicine, Wuhan, China; 4 Hubei Key Laboratory of Theory and Application Research of Liver and Kidney in Traditional Chinese Medicine, Wuhan, China

**Keywords:** adult neurogenesis, metabolic reprogramming, microglial immunometabolism, neurogenic niche, niche remodeling

## Abstract

Adult neurogenesis is dynamically regulated by the neurogenic niche, yet the mechanisms governing its remodeling are not fully elucidated. Current research has increasingly extended beyond the intrinsic properties of neural stem cells to consider the broader microenvironment in which neurogenesis occurs, with microglia emerging as important regulators of the neurogenic niche. Drawing upon pathological contexts such as depression and aging, this review characterizes the hallmarks of neurogenic niche dysregulation and synthesizes the mechanisms by which microglia modulate neurogenic efficiency. Particular emphasis is placed on the role of immunometabolism in shaping microglial functional states and subsequent niche remodeling. We propose that microglial immunometabolic states may serve as an important interface through which pathological perturbations within the brain are linked to remodeling of the neurogenic niche, providing a conceptual framework for understanding neurogenic decline across distinct pathological contexts.

## 1 Introduction

Adult neurogenesis persists in the mammalian subventricular and subgranular zones, though its exact contribution to human brain homeostasis remains a subject of debate (Altman and Das, 1965; Liu et al., 2020). This process involves the activation and differentiation of neural stem cells (NSCs) and the subsequent integration of newly generated neurons into cognitive circuits involved in memory and emotional regulation (Montalbán-Loro et al., 2021; Mazzitelli-Fuentes et al., 2022).

Current paradigms increasingly identify the extrinsic neurogenic niche as an important regulator of adult neurogenesis beyond intrinsic cellular mechanisms. (Lattanzi et al., 2022). Comprising neurons, vasculature, and glia, this niche dictates NSC quiescence, fate specification, and survival (Antonelli et al., 2018). Consequently, fluctuations in neurogenesis often reflect continuous microenvironmental remodeling rather than isolated intrinsic NSC alterations (Zhang et al., 2021).

As resident immune cells, microglia are important regulators of the neurogenic niche, where they contribute to tissue homeostasis and the regulation of neurogenic processes (Gong et al., 2022). The neurogenic niche is a multicellular microenvironment in which microglia interact with neural stem cells, astrocytes, neurons, and other cellular components to influence neurogenesis through inflammatory, trophic, and synaptic signals. Emerging evidence suggests that microglial immunometabolic states are closely linked to their functional regulation (Ciarnelli et al., 2023). These metabolic changes may also contribute to neurogenic niche remodeling by altering the local microenvironment, although this potential link remains to be established.

This review examines the role of microglial immunometabolism in neurogenic niche remodeling, with particular emphasis on depression and aging. We summarize niche alterations in these conditions, discuss the potential mechanisms linking microglial immunometabolism to niche remodeling, and consider their therapeutic implications (Table 1).

**Table 1:** representative studies linking microglial immunometabolism to adult neurogenesis.

References	Experimental model/context	Key finding	Potential implication for the neurogenic niche
Jiao et al. (2026)	Obesity	PPARδ ↑; phagocytosis ↑; inflammation ↓; metabolic flexibility ↑	Relevance to neurogenic niche regulation
Nicolas et al. (2026)	Hippocampal IL-1β-overexpressing rats	Microglial lipid droplets ↑; adult hippocampal neurogenesis ↓	Association with impaired adult neurogenesis
Wang et al. (2024)	LPS-stimulated BV2 cells	Glycolysis ↓; pro-inflammatory responses ↓; M2-like microglial phenotype ↑	Relevance of microglial glycolytic regulation to neurogenic niche function
Yan et al. (2020)	Sepsis-associated brain dysfunction	Mitochondrial function ↑; M2-like microglial response ↑; cognitive impairment ↓	Relevance of microglial mitochondrial remodeling to neurogenic niche function
Luo et al. (2022)	Ischemic stroke	Anti-inflammatory microglial response ↑; neurogenesis ↑; NSC neuronal differentiation ↑	Microglial inflammatory regulation is associated with neurogenic outcomes
Hoshi et al. (2021)	Social defeat stress in mice	Microglial engulfment of NSCs ↑; hippocampal neurogenesis ↓	Microglial engulfment contributes to stress-induced neurogenic impairment
Diaz-Aparicio et al. (2020)	P2Y12- or MerTK/Axl-deficient mice	Microglial phagocytosis ↓; adult hippocampal neurogenesis ↓; neuronal production ↓	Microglial phagocytosis regulates adult hippocampal neurogenesis
Hao et al. (2024)	Depression	Complement C3/CR3 activation ↑; microglial synaptic pruning ↑; synaptic proteins ↓	Relevance to microglia-mediated synaptic remodeling
Madhu et al. (2024)	Alzheimer’s disease	Microglial and astrocytic inflammatory signaling ↓; cognitive and mood function ↑	Relevance of multicellular inflammatory regulation to the neurogenic niche
Nikolopoulos et al. (2023)	Neuropsychiatric lupus with hippocampal dysfunction	Microglial activation ↑; proinflammatory cytokines ↑; hippocampal neurogenesis ↓; IL-6/IL-18-induced hiNSC apoptosis ↑	Microglial inflammatory signaling contributes to impaired adult hippocampal neurogenesis
Zou et al. (2022)	Binge ethanol exposure	Microglial depletion ↓ AHN; pn-EVs ↑ neurogenesis; EtOH-EVs ↓ neurogenesis; G9a/H3K9me2 ↑	Microglial EV signaling regulates adult hippocampal neurogenesis
Zhou et al. (2019)	Ischemic stroke	Microglial JAK2/STAT3 activation ↑; Drp1 phosphorylation and mitochondrial fission ↑; neuroinflammation ↑	Relevance of microglial mitochondrial remodeling to neurogenic niche regulation
Higashi et al. (2026)	Parkinson's disease	Glycolysis and Krebs cycle markers ↓; ketone bodies and amino acid catabolism-related metabolites ↑; phosphatidylcholine (40:2) ↑	Systemic metabolic remodeling in PD may be relevant to metabolic regulation of the neurogenic niche
Chiang and Nicol (2022)	Alzheimer's disease	Oxidative stress, ER stress, and mitochondrial dysfunction ↑; ATP production, mitochondrial respiration, membrane potential, and mitochondrial mass ↓	Aβ-induced metabolic and mitochondrial dysfunction in neural stem cells may contribute to neurogenic niche impairment

## 2 Niche dysregulation in neurogenic disorders

Impaired neurogenesis involves both intrinsic NSC alterations and dysregulation of the neurogenic niche, rather than isolated changes in NSC function (Zamproni et al., 2026). Microglial immunometabolic and functional alterations may contribute to neurogenic niche remodeling in depression and aging.

### 2.1 Inflammatory niche remodeling in depression

Impaired hippocampal neurogenesis has been implicated in the pathophysiology of depression. Chronic stress suppresses neurogenesis in the subgranular zone, whereas neurogenic restoration ameliorates depressive behaviors and cognitive deficits (Anbiao and Jiyan, 2023). This impairment involves global niche dysregulation, in which microglia represent important regulators of the altered microenvironment (He et al., 2024).

Physiologically, microglia preserve niche homeostasis through surveillance, phagocytosis, synaptic pruning, and neurotrophic secretion (Li B. et al., 2022). Chronic stress can disrupt this equilibrium, with studies in CUMS-exposed rats reporting reduced hippocampal brain-derived neurotrophic factor (BDNF) and insulin-like growth factor 1 (IGF-1) expression, elevated hippocampal inflammatory cytokines, and altered microglia-related markers (Wu et al., 2024; Talebi Taheri et al., 2026). Despite phenotypic heterogeneity, microglia in depression may exhibit altered immune-related profiles. Transcriptomic analysis of postmortem brain tissue from individuals with major depressive disorder revealed an immunosuppressive microglial phenotype, accompanied by differential regulation of pathways related to effector functions, including complement signaling and phagocytosis. (Scheepstra et al., 2023).

Microglial metabolic reprogramming involves changes in energy metabolism, including enhanced glycolysis, mitochondrial dysfunction, and elevated oxidative stress, which may reflect altered metabolic adaptation to persistent pathological stimuli. These metabolic changes are associated with altered microglial functional states and may contribute to neurogenic niche remodeling and impaired neurogenesis. (Liu et al., 2025; Dong et al., 2026; Wang et al., 2026). In a mouse model of chronic stress, electroacupuncture promoted BDNF-dependent neurogenesis through microglial reprogramming, highlighting the potential link between changes in microglial functional states and the regulation of adult hippocampal neurogenesis under pathological conditions (Zhang L. et al., 2026).

Postpartum depression may also involve similar mechanisms. Dramatic endocrine and immunological changes during pregnancy and the postpartum period may influence microglial activation and metabolic regulation. Although direct evidence is limited, a study in postpartum C57BL/6J dams exposed to pup separation and chronic restraint stress found that reduced hippocampal inflammation and microglial activation were associated with increased adult hippocampal neurogenesis and stress resilience (Zhou et al., 2025).

### 2.2 Niche exhaustion in brain aging

Aging impairs adult neurogenesis, characterized by reduced NSC activation and diminished production of newborn neurons. These changes are associated with age-related alterations in the intrinsic regulatory mechanisms of NSCs, including increased glucose uptake (Ruetz et al., 2024).

Microglia under pathological conditions like depression often create an inflammatory niche. In contrast, senescent microglia may exhibit a progressive decline in their ability to maintain homeostasis. Their surveillance, migration, and phagocytic functions may become impaired. Consequently, their adaptability to local microenvironmental changes decreases. These changes may progressively compromise their capacity to maintain the neurogenic niche (Wangler and Godbout, 2023; Zhang T. et al., 2026). Importantly, inflammatory changes and impaired homeostatic functions may overlap across these conditions, rather than representing distinct or mutually exclusive patterns of microglial dysfunction.

This functional decline is closely associated with immunometabolic dysregulation. Senescent microglia frequently present with mitochondrial dysfunction, reduced adenosine triphosphate (ATP) production, accumulation of reactive oxygen species (ROS), and decreased metabolic flexibility. They also exhibit abnormal glucose metabolism and lipid droplet accumulation (Hu et al., 2022; Zhao et al., 2024).

Overall, age-related neurogenic decline is associated with progressive impairment of microglial homeostatic function and immunometabolic dysregulation, which may contribute to the deterioration of neurogenic niche support.

**Figure 1:**
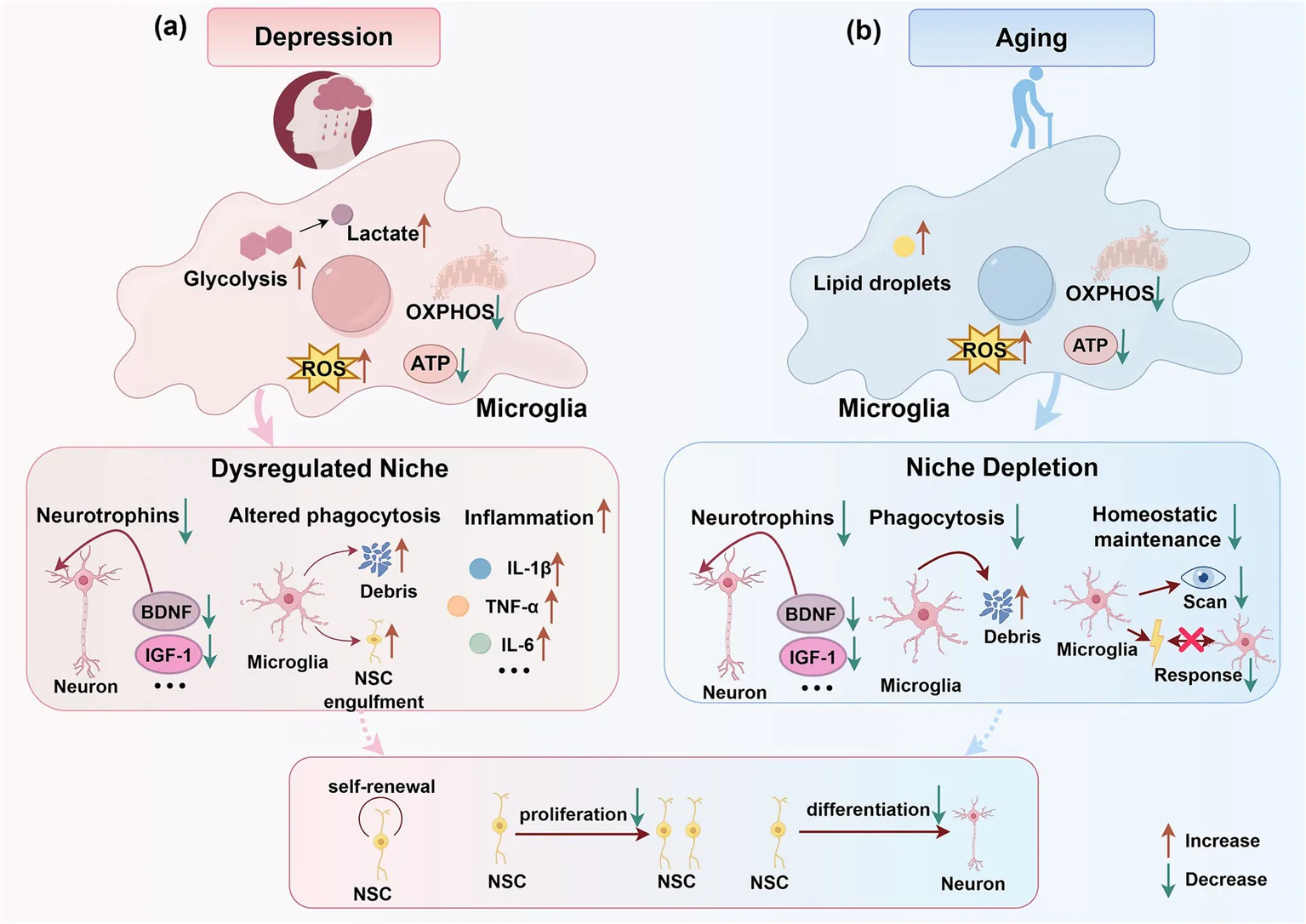
Microglial immunometabolic alterations are associated with neurogenic niche remodeling in depression and aging. **(a)** In depression, enhanced glycolysis, mitochondrial dysfunction, impaired OXPHOS, and oxidative stress in microglia are associated with neurogenic niche dysregulation. **(b)** In aging, microglial lipid accumulation, oxidative stress, and mitochondrial dysfunction are associated with impaired neurogenic niche support. Both conditions may contribute to impaired NSC function. Solid arrows indicate links directly supported by experimental evidence, whereas dashed arrows indicate proposed or indirectly supported links. OXPHOS, oxidative phosphorylation; NSC, neural stem cell.

## 3 Microglial immunometabolic regulation of the neurogenic niche

Microglia regulate the neurogenic niche through coordinated functions, including phagocytosis, synaptic remodeling, neurotrophic signaling, and extracellular vesicle (EV)-mediated communication (Früholz and Meyer-Luehmann, 2024). These functions are closely linked to dynamic and context-dependent microglial immunometabolic states, which exhibit substantial molecular and functional heterogeneity across physiological and pathological conditions (Hamagami et al., 2026). Targeting microglial lipid metabolism in obesity enhanced phagocytosis, reduced inflammatory signaling, and improved metabolic flexibility, although adult neurogenesis was not directly assessed (Jiao et al., 2026). Microglia interact with astrocytes and other cellular populations within the neurogenic niche. In Alzheimer’s disease models, hiPSC-NSC-derived extracellular vesicles were internalized by microglia and neurons and contacted astrocytes (Attaluri et al., 2023). In the same disease model, EV treatment attenuated inflammatory signaling in microglia and astrocytes and improved cognitive and mood function (Madhu et al., 2024).

The following sections examine how microglial immunometabolic states relate to microglial functions, how these functions shape the neurogenic niche, how metabolic and mitochondrial perturbations further remodel its extracellular environment, and how these processes converge within the multicellular niche to influence adult neurogenesis.

### 3.1 Microglial immunometabolic states and functions

Microglial functions are closely coupled to their metabolic state. Under physiological conditions, microglia predominantly rely on mitochondrial oxidative phosphorylation (OXPHOS) to meet the energetic demands of continuous environmental surveillance and homeostatic regulation (Tejera et al., 2019). This metabolic capacity supports processes such as migration, phagocytosis, and intercellular communication (Li Q. et al., 2022). Conversely, chronic hippocampal inflammation impaired exercise-induced neurogenesis and promoted lipid-droplet accumulation in microglia, suggesting an association between microglial metabolic remodeling and altered adult hippocampal neurogenesis (Nicolas et al., 2026).

Pathological stimuli, including chronic inflammation and tissue injury, can induce metabolic reprogramming accompanied by changes in microglial functional states. Enhanced glycolytic activity is generally associated with pro-inflammatory microglial responses, whereas mitochondrial metabolic function is closely linked to microglial functional states and inflammatory regulation (Wang et al., 2022; Yu et al., 2023).However, these associations are not fixed and may vary depending on the stimulus and microglial state. Consistent with this relationship, mitochondrial transplantation promoted a less inflammatory microglial state and improved cognitive impairment in sepsis-associated brain dysfunction, although the underlying metabolic changes were not directly characterized (Yan et al., 2020). Thus, metabolic remodeling is not simply a consequence of altered microglial activity but is closely linked to the functional characteristics through which microglia interact with the neurogenic niche. Changes in microglial functional states can, in turn, be associated with altered neurogenic outcomes. DHLA-AuNC treatment induced a less inflammatory microglial state, and conditioned medium from treated microglia enhanced neurogenesis in neuronal cells and an ex vivo stroke model, although the direct contribution of microglial metabolic remodeling was not assessed (Xiao et al., 2020). In ischemic stroke, repetitive transcranial magnetic stimulation promoted an anti-inflammatory microglial response and enhanced neurogenesis. Conditioned medium from rTMS-treated microglia also promoted neuronal differentiation and reduced apoptosis in NSCs, supporting a link between microglial inflammatory regulation and neurogenic outcomes (Luo et al., 2022).

Several molecular pathways participate in this coupling between metabolism and microglial function. HIF-1α, PI3K/AKT/mTOR, NF-κB, MyD88, and redox-regulatory pathways have been implicated in coordinating metabolic and inflammatory responses in microglia (Cai L. et al., 2024; Vargas et al., 2025; Dai et al., 2026). In LPS-stimulated BV2 cells, Erjingpill bionic cerebrospinal fluid suppressed glycolysis and inflammatory responses through mTOR signaling, although its effects on adult neurogenesis were not directly assessed (Wang et al., 2024). These pathways provide molecular connections between metabolic remodeling and changes in cytokine production, redox signaling, and other microglial activities, thereby providing a mechanistic basis for subsequent niche remodeling.

### 3.2 Immunometabolic regulation of microglial niche functions

The functional consequences of microglial immunometabolic states become evident through their effects on key processes that shape the neurogenic niche, including phagocytosis, synaptic remodeling, and neurotrophic and extracellular vesicle-mediated signaling.

#### 3.2.1 Phagocytosis and niche homeostasis

During adult neurogenesis, a substantial proportion of newborn cells undergoes programmed cell death in early developmental stages. Prompt clearance of these apoptotic cells is paramount for maintaining niche homeostasis. Under physiological conditions, microglia rapidly recognize and engulf dead cells and cellular debris. This rapid clearance prevents the persistent activation of local inflammatory responses (Gray et al., 2020). Notably, microglial phagocytosis extends beyond passive clearance of apoptotic cells. Phagocytosis induces a transcriptional program in microglia that alters their secretome and modulates adult hippocampal neurogenesis (Diaz-Aparicio et al., 2020). In mice subjected to social defeat stress, microglial engulfment of neural stem cells increased and hippocampal neurogenesis was impaired. Both NSC-specific deletion of TRPV4 and pharmacological removal of microglia ameliorated the stress-induced impairment of hippocampal neurogenesis (Hoshi et al., 2021).

These phagocytic functions are metabolically demanding and depend on sufficient cellular bioenergetic capacity. Accordingly, alterations in microglial immunometabolic states may influence the capacity to sustain surveillance, migration, and engulfment, thereby affecting apoptotic cell clearance and the local inflammatory environment.

#### 3.2.2 Synaptic pruning and circuit integration

Neurogenesis encompasses both the generation of newborn neurons and their successful integration into pre-existing neural networks. Microglia participate in neural circuit optimization through activity-dependent synaptic pruning. This process refines network precision and efficiency (Werneburg et al., 2020; Faust et al., 2021). Complement proteins can tag specific synapses for elimination and promote microglial synaptic pruning (Chung et al., 2023). In a mouse model of depression induced by chronic unpredictable mild stress, complement C3/CR3 activation was associated with aberrant microglia-mediated synaptic pruning and reduced expression of synaptic proteins (Hao et al., 2024). Furthermore, the complement system is intricately linked to the neurogenic process itself (Shah et al., 2021). During circuit remodeling driven by the continuous integration of newborn neurons, microglia also facilitate neurogenesis-induced synaptic reorganization (Wang et al., 2020; Huo et al., 2024). Thus, microglia-mediated synaptic remodeling may link neurogenesis with functional circuit reorganization, facilitating the integration of newly generated neurons into functional circuits.

These findings highlight synaptic remodeling as another microglia-mediated process through which changes in microglial functional states can influence the neurogenic niche.

#### 3.2.3 Neurotrophic and extracellular vesicle signaling

Beyond phagocytosis and synaptic pruning, microglia regulate neurogenesis through interactions with neural stem cells and the surrounding niche. Neurotrophic factors, including BDNF and IGF-1, are involved in the regulation of neurogenesis (Wang et al., 2017). Conversely, persistent microglial activation and pro-inflammatory cytokine production can be associated with impaired hippocampal neurogenesis. In lupus-prone mice modeling neuropsychiatric lupus, microglial activation and increased pro-inflammatory cytokines were accompanied by disrupted hippocampal neurogenesis and increased apoptosis of adult neural stem cells (Nikolopoulos et al., 2023). Microglia-induced neuroinflammation has also been implicated in impaired hippocampal neurogenesis following traumatic brain injury (Navabi et al., 2024).

The metabolic state of microglia is also linked to their trophic functions. Mitochondrial dysfunction has been associated with reduced expression of IGF-1 and BDNF, whereas promotion of mitochondrial biogenesis can enhance markers associated with reparative microglial states and increase BDNF secretion (Ferger et al., 2010; Ma et al., 2018; Pomierny et al., 2021). These findings suggest that mitochondrial metabolic integrity contributes to the capacity of microglia to provide trophic support within the neurogenic niche.

In addition to classical neurotrophic factors, microglia utilize EVs for intercellular communication within the neurogenic niche. By delivering nucleic acids, proteins, and metabolism-related molecules, they participate in NSC fate determination, glial crosstalk, and local microenvironmental regulation (Fan et al., 2022; Xu et al., 2026). Under physiological conditions, microglia secrete pro-neurogenic extracellular vesicles that support adult hippocampal neurogenesis. Under pathological conditions, the molecular cargo of microglial EVs can be altered, resulting in impaired neurogenesis. In an ethanol exposure model, pro-inflammatory microglial EVs were enriched in G9a mRNA, increased G9a/H3K9me2 levels in recipient cells, and impaired adult hippocampal neurogenesis (Zou et al., 2022).

Together, these findings indicate that microglial immunometabolic states can influence trophic support and EV-mediated communication, extending the effects of intracellular metabolic changes to the surrounding neurogenic niche. Such metabolic regulation may therefore contribute to broader changes in intercellular signaling and niche homeostasis.

### 3.3 Metabolic and mitochondrial remodeling of the neurogenic niche

While microglial immunometabolic states shape microglial functions, their effects may extend further to the extracellular environment of the neurogenic niche. Metabolic and mitochondrial disturbances can alter this environment and thereby influence the neural stem and progenitor cells that sustain adult neurogenesis. Mitochondrial dysfunction is increasingly recognized as an important contributor to pathological niche remodeling. Dynamin-related protein 1 (Drp1), a key regulator of mitochondrial fission, is closely associated with microglial inflammatory responses. In an ischemic stroke model, inhibition of JAK2/STAT3 signaling reduced Drp1 phosphorylation, mitochondrial translocation, and fission in microglia, accompanied by reduced neuroinflammation (Zhou et al., 2019). The role of Drp1 in regulating microglial inflammatory responses and its relationship with mitochondrial dysfunction have also been discussed in neurodegenerative diseases (Cai P. et al., 2024). Furthermore, reverse electron transport mediated by mitochondrial complex I accelerates ROS generation, contributing to neuroinflammation (Peruzzotti-Jametti et al., 2024). Excessive ROS not only inflicts direct damage upon neural tissue but also impairs neurogenesis by inhibiting NSC proliferation and differentiation (Correia et al., 2023).

These findings suggest that mitochondrial perturbation may link intracellular metabolic dysfunction with changes in the neurogenic environment through oxidative stress and related cellular responses. In an Alzheimer’s disease-related experimental model, amyloid-beta (Aβ) induced oxidative stress, endoplasmic reticulum stress, and mitochondrial dysfunction in human neural stem cells, including impaired ATP production and mitochondrial respiration (Chiang and Nicol, 2022). Although these findings do not directly establish how microglial immunometabolism affects adult neurogenesis, they demonstrate that Alzheimer’s disease-related pathology can impair the metabolic and mitochondrial functions of neural stem cells, potentially contributing to an altered neurogenic environment.

Beyond redox signaling, metabolic intermediates may provide additional routes for communication between microglia and the surrounding niche. Intermediary metabolites such as lactate, succinate, and ketone bodies can function as signaling molecules by modulating redox states, epigenetic modifications, and cellular signaling pathways (Ristic et al., 2017; Wang et al., 2025). Metabolomic studies have also identified systemic metabolic alterations in neurological disorders, including reduced glycolytic and Krebs cycle intermediates and increased ketone bodies and amino acid metabolism-related metabolites in patients with Parkinson’s disease (Higashi et al., 2026). However, direct evidence that microglia-derived metabolites regulate adult neurogenesis remains limited. Their potential contribution to niche remodeling therefore remains an important area for further investigation.

Overall, mitochondrial dysfunction, oxidative stress, and altered metabolite signaling may contribute to the remodeling of the neurogenic niche in association with microglial immunometabolic disturbances.

### 3.4 Multicellular immunometabolic remodeling of the neurogenic niche

The effects of microglial immunometabolism on the neurogenic niche extend beyond individual microglial functions. Microglia interact with astrocytes, neural stem cells, neurons, and other cellular components of the niche, creating a dynamic environment in which inflammatory, trophic, metabolic, and extracellular signaling processes are closely interconnected. Taken together, the findings discussed above suggest that alterations in microglial immunometabolic states can reshape interactions among niche cells through multiple interconnected processes rather than a single linear pathway.

Importantly, available evidence does not establish a single linear pathway linking a defined microglial metabolic state to a specific neurogenic outcome across physiological and pathological conditions. Instead, microglial immunometabolism may represent an important regulatory interface linking microglial functional states with multicellular niche remodeling. Pathological perturbations within the brain may alter microglial metabolic states and associated functional and intercellular signaling processes, thereby contributing to niche remodeling and altered adult neurogenesis.

**Figure 2:**
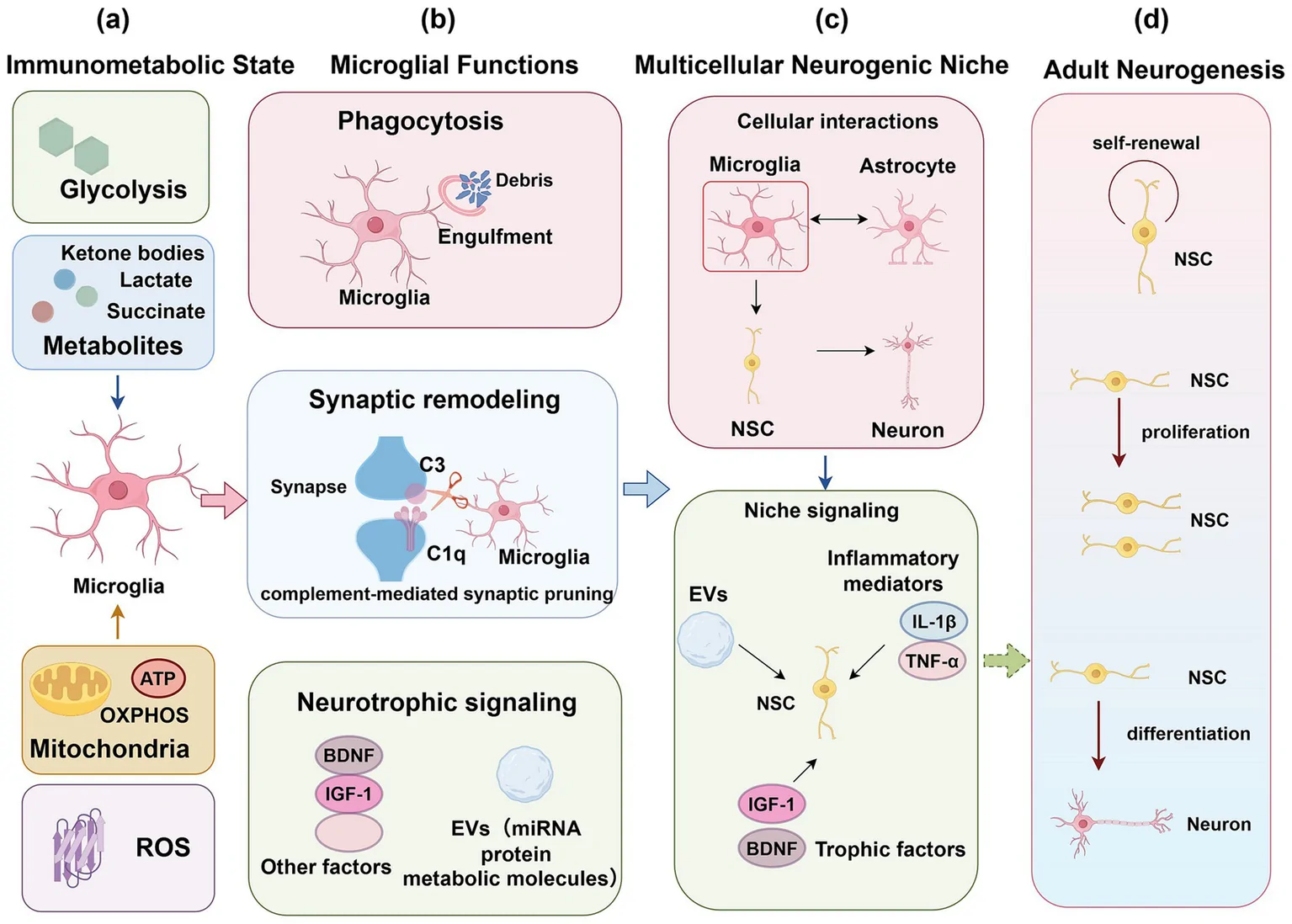
**(a)** Microglial immunometabolic states are characterized by changes in glycolysis, mitochondrial function, ROS production, and metabolic intermediates. **(b)** These immunometabolic states are associated with microglial functional outputs, including phagocytosis, synaptic remodeling, and neurotrophic signaling. **(c)** These functions operate within a multicellular neurogenic niche involving microglia, astrocytes, NSCs, and neurons, together with inflammatory mediators, trophic factors, and EV-mediated signaling. **(d)** Alterations in the neurogenic niche may affect adult neurogenesis, including NSC self-renewal, proliferation, neuronal differentiation, and neuronal integration. Solid arrows indicate links directly supported by experimental evidence, whereas dashed arrows indicate proposed or indirectly supported links. NSC, neural stem cell; EV, extracellular vesicle; ROS, reactive oxygen species.

## 4 Targeting microglial immunometabolism

Targeting microglial immunometabolism to restore homeostatic function may represent a potential strategy for ameliorating neurogenesis deficits.

Improving mitochondrial function is an important strategy for intervening in microglial immunometabolism. Enhancing mitochondrial biogenesis, balancing mitochondrial dynamics, or promoting the clearance of damaged mitochondria can suppress excessive microglial inflammatory activation. Concurrently, these interventions enhance neurotrophic support. Furthermore, exogenous mitochondrial transplantation offers a novel approach. Microglia can internalize these exogenous mitochondria, leading to elevated ATP levels and reduced ROS accumulation (Sun et al., 2024).

Metabolic sensing pathways provide critical nodes for modulating microglial immunometabolic states. Signaling networks involving AMPK, mTOR, SIRT1, and PGC-1α integrate cellular energy status with inflammatory responses. They consequently shape microglial metabolic states and functional outputs (Li et al., 2024; Moon et al., 2026). Modulating these pathways can mitigate inflammatory activation, improve mitochondrial function, and augment neurotrophic support.

Niche interactive regulation emphasizes the reconstruction of communication networks between NSCs and microglia. Studies reveal that microglia-derived exosomes can deliver pro-neurogenic miRNAs to promote NSC differentiation into neurons. They also enhance neural regeneration at injury sites. This suggests that targeting intercellular communication networks may facilitate neurogenic niche remodeling (Ruan et al., 2023).

## 5 Conclusion

Adult neurogenesis is a dynamic process shaped by multicellular interactions within the niche microenvironment, involving microglia, neural stem cells, astrocytes, neurons, and other cellular components. The extrinsic niche represents an important regulatory component of neurogenic efficiency alongside intrinsic cellular programs. As central regulators of this milieu, microglia influence neurogenic outcomes through functional states that contribute to microenvironmental homeostasis.

The synthesis of current evidence highlights immunometabolism as an important link between microglial activity and niche remodeling. This immunometabolic framework may help contextualize niche exhaustion and neurogenic decline across pathological contexts such as aging and depression. Consequently, microglial metabolic states may represent an important mechanism through which systemic perturbations are linked to localized neurogenic deficits.

Deciphering the spatiotemporal dynamics of microglial metabolic subpopulations remains a critical challenge. Future investigations utilizing spatial transcriptomics and advanced metabolic profiling will be essential to elucidate these regulatory mechanisms across diverse pathological landscapes. Such approaches may facilitate the development of precision therapeutic strategies aimed at restoring the neurogenic niche and improving neurogenesis.
